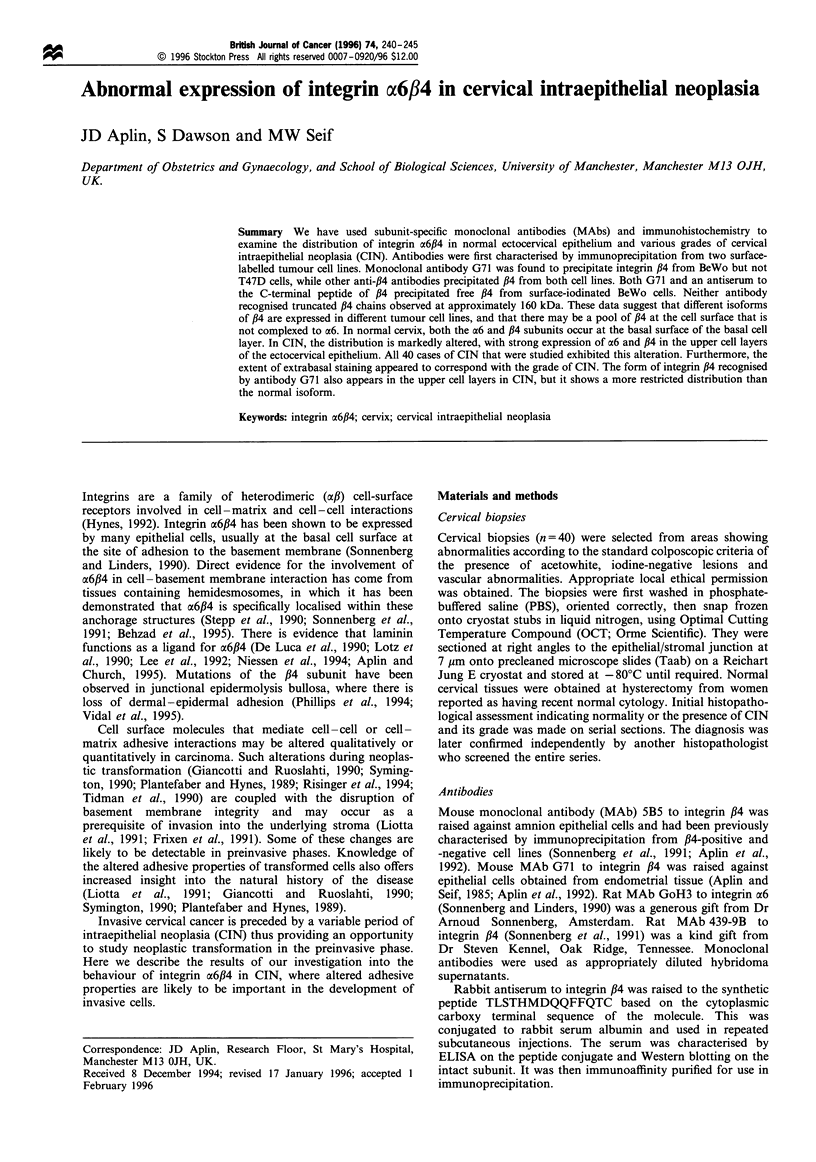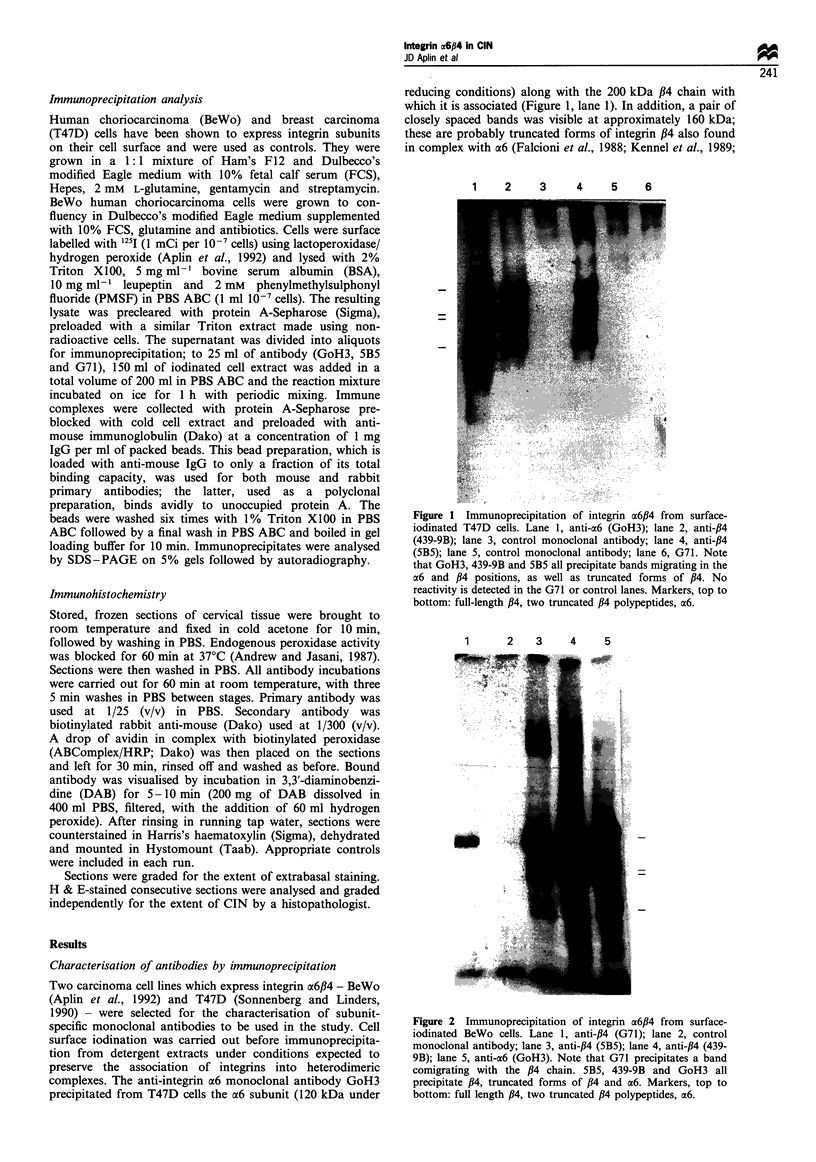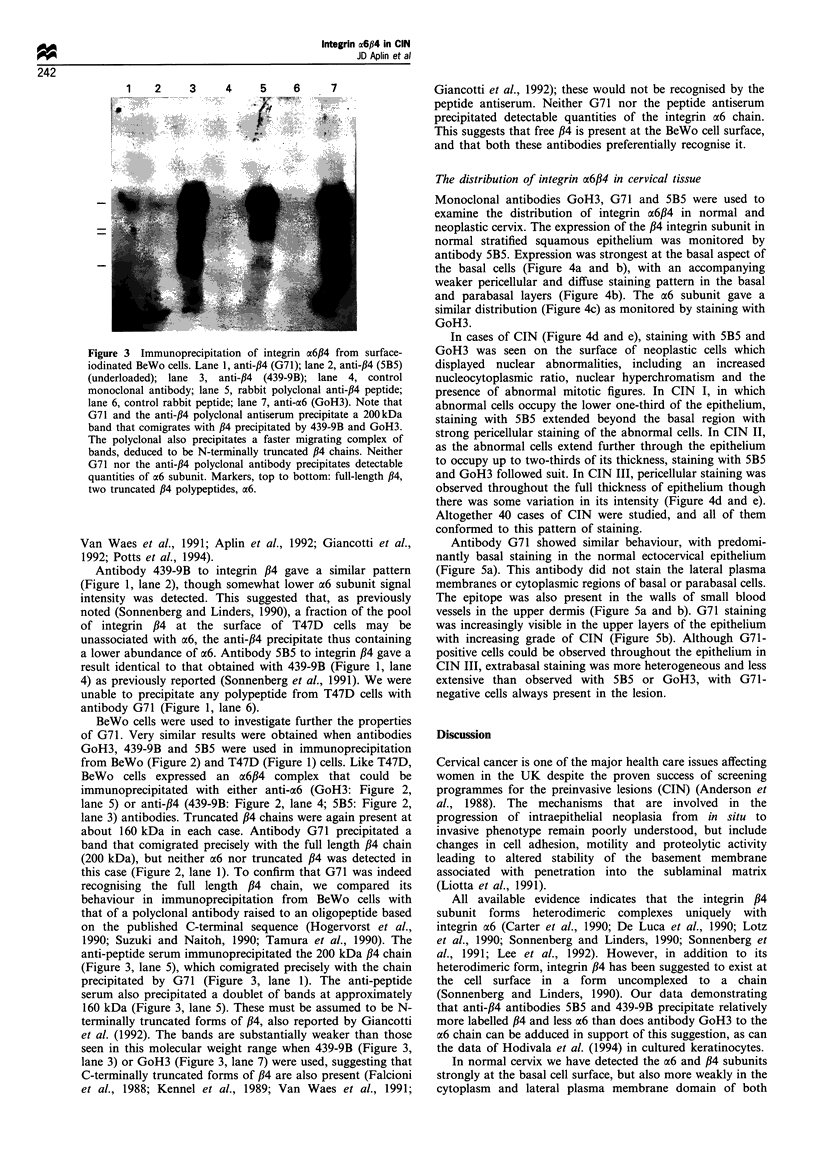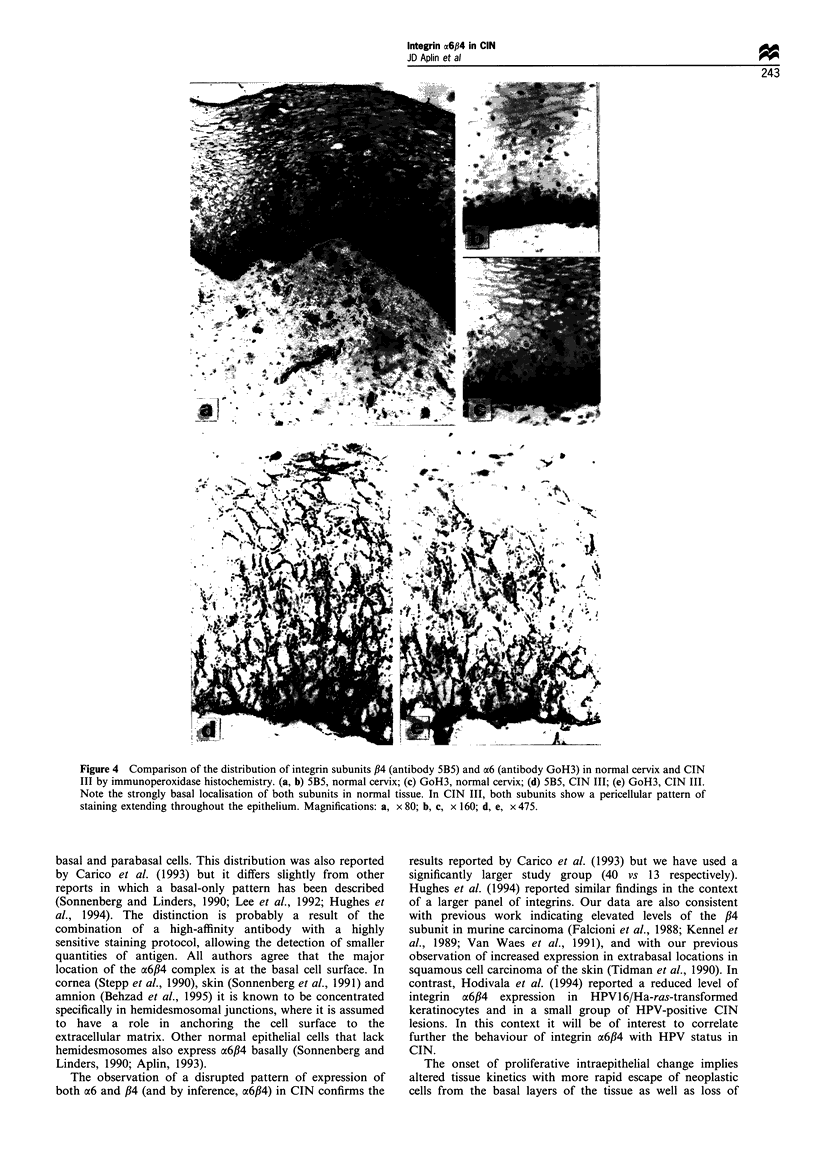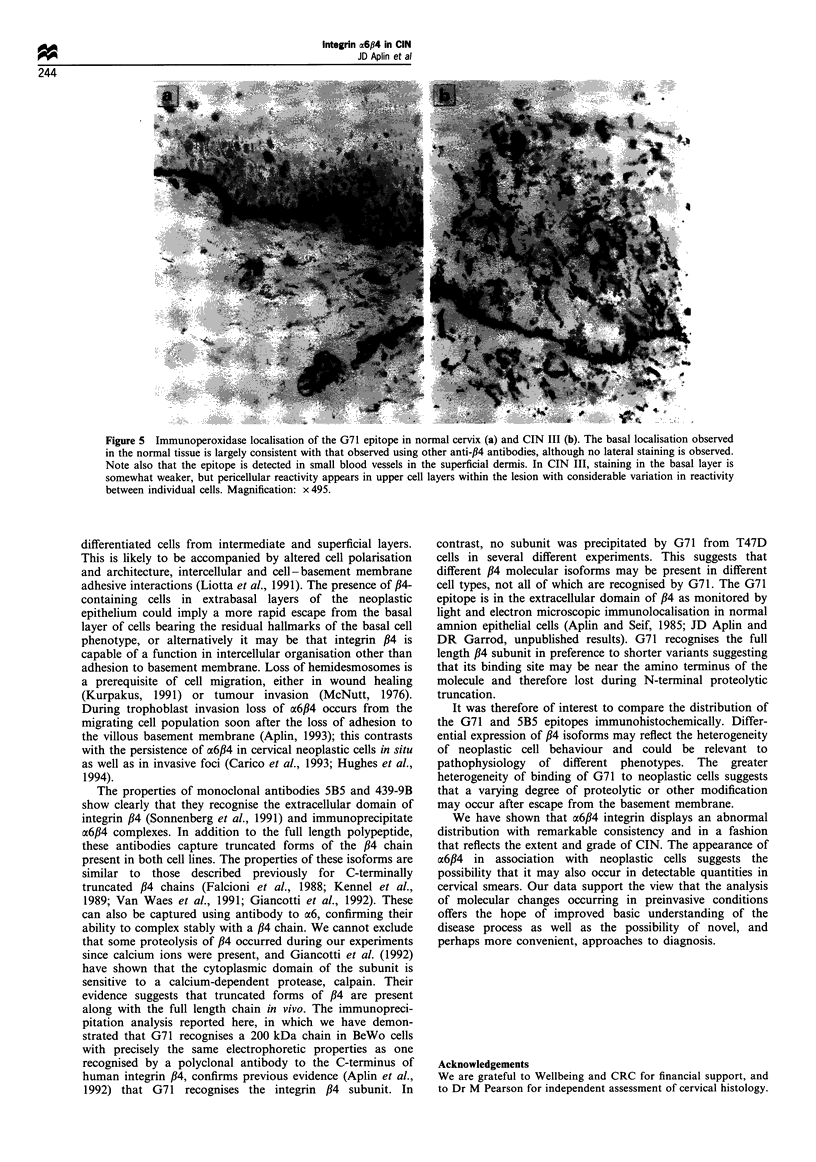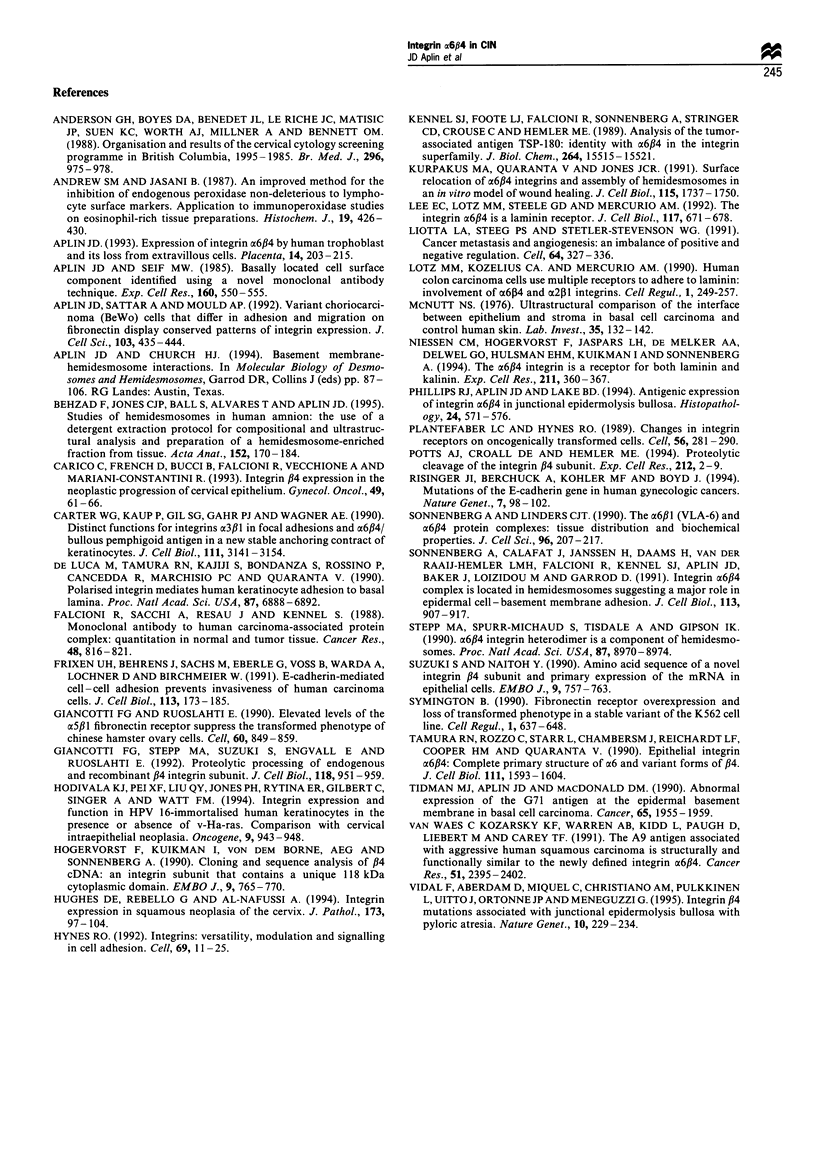# Abnormal expression of integrin alpha 6 beta 4 in cervical intraepithelial neoplasia.

**DOI:** 10.1038/bjc.1996.344

**Published:** 1996-07

**Authors:** J. D. Aplin, S. Dawson, M. W. Seif

**Affiliations:** Department of Obstetrics and Gynaecology, University of Manchester, UK.

## Abstract

**Images:**


British Journal of Cancer (1996) 74, 240-245
© 1996 Stockton Press All rights reserved 0007-0920/96 $12.00

### Abnormal expression of integrin α6β4 in cervical intraepithelial neoplasia

JD Aplin, S Dawson and MW Seif

*Department of Obstetrics and Gynaecology, and School of Biological Sciences, University of Manchester, Manchester M13 OJH, UK.*

**Summary** We have used subunit-specific monoclonal antibodies (MAbs) and immunohistochemistry to examine the distribution of integrin α6β4 in normal ectocervical epithelium and various grades of cervical intraepithelial neoplasia (CIN). Antibodies were first characterised by immunoprecipitation from two surface-labelled tumour cell lines. Monoclonal antibody G71 was found to precipitate integrin β4 from BeWo but not T47D cells, while other anti-β4 antibodies precipitated β4 from both cell lines. Both G71 and an antiserum to the C-terminal peptide of β4 precipitated free β4 from surface-iodinated BeWo cells. Neither antibody recognised truncated β4 chains observed at approximately 160 kDa. These data suggest that different isoforms of β4 are expressed in different tumour cell lines, and that there may be a pool of β4 at the cell surface that is not complexed to α6. In normal cervix, both the α6 and β4 subunits occur at the basal surface of the basal cell layer. In CIN, the distribution is markedly altered, with strong expression of α6 and β4 in the upper cell layers of the ectocervical epithelium. All 40 cases of CIN that were studied exhibited this alteration. Furthermore, the extent of extrabasal staining appeared to correspond with the grade of CIN. The form of integrin β4 recognised by antibody G71 also appears in the upper cell layers in CIN, but it shows a more restricted distribution than the normal isoform.

**Keywords:** integrin α6β4; cervix; cervical intraepithelial neoplasia

Integrins are a family of heterodimeric (αβ) cell-surface receptors involved in cell–matrix and cell–cell interactions (Hynes, 1992). Integrin α6β4 has been shown to be expressed by many epithelial cells, usually at the basal cell surface at the site of adhesion to the basement membrane (Sonnenberg and Linders, 1990). Direct evidence for the involvement of α6β4 in cell–basement membrane interaction has come from tissues containing hemidesmosomes, in which it has been demonstrated that α6β4 is specifically localised within these anchorage structures (Stepp *et al.*, 1990; Sonnenberg *et al.*, 1991; Behzad *et al.*, 1995). There is evidence that laminin functions as a ligand for α6β4 (De Luca *et al.*, 1990; Lotz *et al.*, 1990; Lee *et al.*, 1992; Niessen *et al.*, 1994; Aplin and Church, 1995). Mutations of the β4 subunit have been observed in junctional epidermolysis bullosa, where there is loss of dermal–epidermal adhesion (Phillips *et al.*, 1994; Vidal *et al.*, 1995).

Cell surface molecules that mediate cell–cell or cell–matrix adhesive interactions may be altered qualitatively or quantitatively in carcinoma. Such alterations during neoplastic transformation (Giancotti and Ruoslahti, 1990; Symington, 1990; Plantefaber and Hynes, 1989; Risinger *et al.*, 1994; Tidman *et al.*, 1990) are coupled with the disruption of basement membrane integrity and may occur as a prerequisite of invasion into the underlying stroma (Liotta *et al.*, 1991; Frixen *et al.*, 1991). Some of these changes are likely to be detectable in preinvasive phases. Knowledge of the altered adhesive properties of transformed cells also offers increased insight into the natural history of the disease (Liotta *et al.*, 1991; Giancotti and Ruoslahti, 1990; Symington, 1990; Plantefaber and Hynes, 1989).

Invasive cervical cancer is preceded by a variable period of intraepithelial neoplasia (CIN) thus providing an opportunity to study neoplastic transformation in the preinvasive phase. Here we describe the results of our investigation into the behaviour of integrin α6β4 in CIN, where altered adhesive properties are likely to be important in the development of invasive cells.

Correspondence: JD Aplin, Research Floor, St Mary's Hospital, Manchester M13 0JH, UK.
Received 8 December 1994; revised 17 January 1996; accepted 1 February 1996

### Materials and methods

### *Cervical biopsies*

Cervical biopsies ($n=40$) were selected from areas showing abnormalities according to the standard colposcopic criteria of the presence of acetowhite, iodine-negative lesions and vascular abnormalities. Appropriate local ethical permission was obtained. The biopsies were first washed in phosphate-buffered saline (PBS), oriented correctly, then snap frozen onto cryostat stubs in liquid nitrogen, using Optimal Cutting Temperature Compound (OCT; Orme Scientific). They were sectioned at right angles to the epithelial/stromal junction at 7 μm onto precleaned microscope slides (Taab) on a Reichart Jung E cryostat and stored at −80°C until required. Normal cervical tissues were obtained at hysterectomy from women reported as having recent normal cytology. Initial histopathological assessment indicating normality or the presence of CIN and its grade was made on serial sections. The diagnosis was later confirmed independently by another histopathologist who screened the entire series.

### *Antibodies*

Mouse monoclonal antibody (MAb) 5B5 to integrin β4 was raised against amnion epithelial cells and had been previously characterised by immunoprecipitation from β4-positive and -negative cell lines (Sonnenberg *et al.*, 1991; Aplin *et al.*, 1992). Mouse MAb G71 to integrin β4 was raised against epithelial cells obtained from endometrial tissue (Aplin and Seif, 1985; Aplin *et al.*, 1992). Rat MAb GoH3 to integrin α6 (Sonnenberg and Linders, 1990) was a generous gift from Dr Arnoud Sonnenberg, Amsterdam. Rat MAb 439-9B to integrin β4 (Sonnenberg *et al.*, 1991) was a kind gift from Dr Steven Kennel, Oak Ridge, Tennessee. Monoclonal antibodies were used as appropriately diluted hybridoma supernatants.

Rabbit antiserum to integrin β4 was raised to the synthetic peptide TLSTHMDQQFFQTC based on the cytoplasmic carboxy terminal sequence of the molecule. This was conjugated to rabbit serum albumin and used in repeated subcutaneous injections. The serum was characterised by ELISA on the peptide conjugate and Western blotting on the intact subunit. It was then immunoaffinity purified for use in immunoprecipitation.

*Immunoprecipitation analysis*

Human choriocarcinoma (BeWo) and breast carcinoma (T47D) cells have been shown to express integrin subunits on their cell surface and were used as controls. They were grown in a 1:1 mixture of Ham's F12 and Dulbecco's modified Eagle medium with 10% fetal calf serum (FCS), Hepes, 2 mM L-glutamine, gentamycin and streptamycin. BeWo human choriocarcinoma cells were grown to confluency in Dulbecco's modified Eagle medium supplemented with 10% FCS, glutamine and antibiotics. Cells were surface labelled with $^{125}I$ (1 mCi per $10^{-7}$ cells) using lactoperoxidase/hydrogen peroxide (Aplin *et al.*, 1992) and lysed with 2% Triton X100, 5 mg $ml^{-1}$ bovine serum albumin (BSA), 10 mg $ml^{-1}$ leupeptin and 2 mM phenylmethylsulphonyl fluoride (PMSF) in PBS ABC (1 ml $10^{-7}$ cells). The resulting lysate was precleared with protein A-Sepharose (Sigma), preloaded with a similar Triton extract made using non-radioactive cells. The supernatant was divided into aliquots for immunoprecipitation; to 25 ml of antibody (GoH3, 5B5 and G71), 150 ml of iodinated cell extract was added in a total volume of 200 ml in PBS ABC and the reaction mixture incubated on ice for 1 h with periodic mixing. Immune complexes were collected with protein A-Sepharose pre-blocked with cold cell extract and preloaded with anti-mouse immunoglobulin (Dako) at a concentration of 1 mg IgG per ml of packed beads. This bead preparation, which is loaded with anti-mouse IgG to only a fraction of its total binding capacity, was used for both mouse and rabbit primary antibodies; the latter, used as a polyclonal preparation, binds avidly to unoccupied protein A. The beads were washed six times with 1% Triton X100 in PBS ABC followed by a final wash in PBS ABC and boiled in gel loading buffer for 10 min. Immunoprecipitates were analysed by SDS–PAGE on 5% gels followed by autoradiography.

*Immunohistochemistry*

Stored, frozen sections of cervical tissue were brought to room temperature and fixed in cold acetone for 10 min, followed by washing in PBS. Endogenous peroxidase activity was blocked for 60 min at 37°C (Andrew and Jasani, 1987). Sections were then washed in PBS. All antibody incubations were carried out for 60 min at room temperature, with three 5 min washes in PBS between stages. Primary antibody was used at 1/25 (v/v) in PBS. Secondary antibody was biotinylated rabbit anti-mouse (Dako) used at 1/300 (v/v). A drop of avidin in complex with biotinylated peroxidase (ABComplex/HRP; Dako) was then placed on the sections and left for 30 min, rinsed off and washed as before. Bound antibody was visualised by incubation in 3,3′-diaminobenzidine (DAB) for 5–10 min (200 mg of DAB dissolved in 400 ml PBS, filtered, with the addition of 60 ml hydrogen peroxide). After rinsing in running tap water, sections were counterstained in Harris's haematoxylin (Sigma), dehydrated and mounted in Hystomount (Taab). Appropriate controls were included in each run.

Sections were graded for the extent of extrabasal staining. H & E-stained consecutive sections were analysed and graded independently for the extent of CIN by a histopathologist.

### Results

*Characterisation of antibodies by immunoprecipitation*

Two carcinoma cell lines which express integrin α6β4 – BeWo (Aplin *et al.*, 1992) and T47D (Sonnenberg and Linders, 1990) – were selected for the characterisation of subunit-specific monoclonal antibodies to be used in the study. Cell surface iodination was carried out before immunoprecipitation from detergent extracts under conditions expected to preserve the association of integrins into heterodimeric complexes. The anti-integrin α6 monoclonal antibody GoH3 precipitated from T47D cells the α6 subunit (120 kDa under reducing conditions) along with the 200 kDa β4 chain with which it is associated (Figure 1, lane 1). In addition, a pair of closely spaced bands was visible at approximately 160 kDa; these are probably truncated forms of integrin β4 also found in complex with α6 (Falcioni *et al.*, 1988; Kennel *et al.*, 1989;

**Figure 1** Immunoprecipitation of integrin α6β4 from surface-iodinated T47D cells. Lane 1, anti-α6 (GoH3); lane 2, anti-β4 (439-9B); lane 3, control monoclonal antibody; lane 4, anti-β4 (5B5); lane 5, control monoclonal antibody; lane 6, G71. Note that GoH3, 439-9B and 5B5 all precipitate bands migrating in the α6 and β4 positions, as well as truncated forms of β4. No reactivity is detected in the G71 or control lanes. Markers, top to bottom: full-length β4, two truncated β4 polypeptides, α6.

**Figure 2** Immunoprecipitation of integrin α6β4 from surface-iodinated BeWo cells. Lane 1, anti-β4 (G71); lane 2, control monoclonal antibody; lane 3, anti-β4 (5B5); lane 4, anti-β4 (439-9B); lane 5, anti-α6 (GoH3). Note that G71 precipitates a band comigrating with the β4 chain. 5B5, 439-9B and GoH3 all precipitate β4, truncated forms of β4 and α6. Markers, top to bottom: full length β4, two truncated β4 polypeptides, α6.

**Figure 3** Immunoprecipitation of integrin α6β4 from surface-iodinated BeWo cells. Lane 1, anti-β4 (G71); lane 2, anti-β4 (5B5) (underloaded); lane 3, anti-β4 (439-9B); lane 4, control monoclonal antibody; lane 5, rabbit polyclonal anti-β4 peptide; lane 6, control rabbit peptide; lane 7, anti-α6 (GoH3). Note that G71 and the anti-β4 polyclonal antiserum precipitate a 200 kDa band that comigrates with β4 precipitated by 439-9B and GoH3. The polyclonal also precipitates a faster migrating complex of bands, deduced to be N-terminally truncated β4 chains. Neither G71 nor the anti-β4 polyclonal antibody precipitates detectable quantities of α6 subunit. Markers, top to bottom: full-length β4, two truncated β4 polypeptides, α6.

Van Waes *et al*., 1991; Aplin *et al*., 1992; Giancotti *et al*., 1992; Potts *et al*., 1994).

Antibody 439-9B to integrin β4 gave a similar pattern (Figure 1, lane 2), though somewhat lower α6 subunit signal intensity was detected. This suggested that, as previously noted (Sonnenberg and Linders, 1990), a fraction of the pool of integrin β4 at the surface of T47D cells may be unassociated with α6, the anti-β4 precipitate thus containing a lower abundance of α6. Antibody 5B5 to integrin β4 gave a result identical to that obtained with 439-9B (Figure 1, lane 4) as previously reported (Sonnenberg *et al*., 1991). We were unable to precipitate any polypeptide from T47D cells with antibody G71 (Figure 1, lane 6).

BeWo cells were used to investigate further the properties of G71. Very similar results were obtained when antibodies GoH3, 439-9B and 5B5 were used in immunoprecipitation from BeWo (Figure 2) and T47D (Figure 1) cells. Like T47D, BeWo cells expressed an α6β4 complex that could be immunoprecipitated with either anti-α6 (GoH3: Figure 2, lane 5) or anti-β4 (439-9B: Figure 2, lane 4; 5B5: Figure 2, lane 3) antibodies. Truncated β4 chains were again present at about 160 kDa in each case. Antibody G71 precipitated a band that comigrated precisely with the full length β4 chain (200 kDa), but neither α6 nor truncated β4 was detected in this case (Figure 2, lane 1). To confirm that G71 was indeed recognising the full length β4 chain, we compared its behaviour in immunoprecipitation from BeWo cells with that of a polyclonal antibody raised to an oligopeptide based on the published C-terminal sequence (Hogervorst *et al*., 1990; Suzuki and Naitoh, 1990; Tamura *et al*., 1990). The anti-peptide serum immunoprecipitated the 200 kDa β4 chain (Figure 3, lane 5), which comigrated precisely with the chain precipitated by G71 (Figure 3, lane 1). The anti-peptide serum also precipitated a doublet of bands at approximately 160 kDa (Figure 3, lane 5). These must be assumed to be N-terminally truncated forms of β4, also reported by Giancotti *et al*. (1992). The bands are substantially weaker than those seen in this molecular weight range when 439-9B (Figure 3, lane 3) or GoH3 (Figure 3, lane 7) were used, suggesting that C-terminally truncated forms of β4 are also present (Falcioni *et al*., 1988; Kennel *et al*., 1989; Van Waes *et al*., 1991; Giancotti *et al*., 1992); these would not be recognised by the peptide antiserum. Neither G71 nor the peptide antiserum precipitated detectable quantities of the integrin α6 chain. This suggests that free β4 is present at the BeWo cell surface, and that both these antibodies preferentially recognise it.

### *The distribution of integrin α6β4 in cervical tissue*

Monoclonal antibodies GoH3, G71 and 5B5 were used to examine the distribution of integrin α6β4 in normal and neoplastic cervix. The expression of the β4 integrin subunit in normal stratified squamous epithelium was monitored by antibody 5B5. Expression was strongest at the basal aspect of the basal cells (Figure 4a and b), with an accompanying weaker pericellular and diffuse staining pattern in the basal and parabasal layers (Figure 4b). The α6 subunit gave a similar distribution (Figure 4c) as monitored by staining with GoH3.

In cases of CIN (Figure 4d and e), staining with 5B5 and GoH3 was seen on the surface of neoplastic cells which displayed nuclear abnormalities, including an increased nucleocytoplasmic ratio, nuclear hyperchromatism and the presence of abnormal mitotic figures. In CIN I, in which abnormal cells occupy the lower one-third of the epithelium, staining with 5B5 extended beyond the basal region with strong pericellular staining of the abnormal cells. In CIN II, as the abnormal cells extend further through the epithelium to occupy up to two-thirds of its thickness, staining with 5B5 and GoH3 followed suit. In CIN III, pericellular staining was observed throughout the full thickness of epithelium though there was some variation in its intensity (Figure 4d and e). Altogether 40 cases of CIN were studied, and all of them conformed to this pattern of staining.

Antibody G71 showed similar behaviour, with predominantly basal staining in the normal ectocervical epithelium (Figure 5a). This antibody did not stain the lateral plasma membranes or cytoplasmic regions of basal or parabasal cells. The epitope was also present in the walls of small blood vessels in the upper dermis (Figure 5a and b). G71 staining was increasingly visible in the upper layers of the epithelium with increasing grade of CIN (Figure 5b). Although G71-positive cells could be observed throughout the epithelium in CIN III, extrabasal staining was more heterogeneous and less extensive than observed with 5B5 or GoH3, with G71-negative cells always present in the lesion.

### Discussion

Cervical cancer is one of the major health care issues affecting women in the UK despite the proven success of screening programmes for the preinvasive lesions (CIN) (Anderson *et al*., 1988). The mechanisms that are involved in the progression of intraepithelial neoplasia from *in situ* to invasive phenotype remain poorly understood, but include changes in cell adhesion, motility and proteolytic activity leading to altered stability of the basement membrane associated with penetration into the sublaminal matrix (Liotta *et al*., 1991).

All available evidence indicates that the integrin β4 subunit forms heterodimeric complexes uniquely with integrin α6 (Carter *et al*., 1990; De Luca *et al*., 1990; Lotz *et al*., 1990; Sonnenberg and Linders, 1990; Sonnenberg *et al*., 1991; Lee *et al*., 1992). However, in addition to its heterodimeric form, integrin β4 has been suggested to exist at the cell surface in a form uncomplexed to a chain (Sonnenberg and Linders, 1990). Our data demonstrating that anti-β4 antibodies 5B5 and 439-9B precipitate relatively more labelled β4 and less α6 than does antibody GoH3 to the α6 chain can be adduced in support of this suggestion, as can the data of Hodivala *et al*. (1994) in cultured keratinocytes.

In normal cervix we have detected the α6 and β4 subunits strongly at the basal cell surface, but also more weakly in the cytoplasm and lateral plasma membrane domain of both

**Figure 4** Comparison of the distribution of integrin subunits β4 (antibody 5B5) and α6 (antibody GoH3) in normal cervix and CIN III by immunoperoxidase histochemistry. (**a**, **b**) 5B5, normal cervix; (**c**) GoH3, normal cervix; (**d**) 5B5, CIN III; (**e**) GoH3, CIN III. Note the strongly basal localisation of both subunits in normal tissue. In CIN III, both subunits show a pericellular pattern of staining extending throughout the epithelium. Magnifications: **a**, ×80; **b**, **c**, ×160; **d**, **e**, ×475.

basal and parabasal cells. This distribution was also reported by Carico *et al.* (1993) but it differs slightly from other reports in which a basal-only pattern has been described (Sonnenberg and Linders, 1990; Lee *et al.*, 1992; Hughes *et al.*, 1994). The distinction is probably a result of the combination of a high-affinity antibody with a highly sensitive staining protocol, allowing the detection of smaller quantities of antigen. All authors agree that the major location of the α6β4 complex is at the basal cell surface. In cornea (Stepp *et al.*, 1990), skin (Sonnenberg *et al.*, 1991) and amnion (Behzad *et al.*, 1995) it is known to be concentrated specifically in hemidesmosomal junctions, where it is assumed to have a role in anchoring the cell surface to the extracellular matrix. Other normal epithelial cells that lack hemidesmosomes also express α6β4 basally (Sonnenberg and Linders, 1990; Aplin, 1993).

The observation of a disrupted pattern of expression of both α6 and β4 (and by inference, α6β4) in CIN confirms the results reported by Carico *et al.* (1993) but we have used a significantly larger study group (40 *vs* 13 respectively). Hughes *et al.* (1994) reported similar findings in the context of a larger panel of integrins. Our data are also consistent with previous work indicating elevated levels of the β4 subunit in murine carcinoma (Falcioni *et al.*, 1988; Kennel *et al.*, 1989; Van Waes *et al.*, 1991), and with our previous observation of increased expression in extrabasal locations in squamous cell carcinoma of the skin (Tidman *et al.*, 1990). In contrast, Hodivala *et al.* (1994) reported a reduced level of integrin α6β4 expression in HPV16/Ha-*ras*-transformed keratinocytes and in a small group of HPV-positive CIN lesions. In this context it will be of interest to correlate further the behaviour of integrin α6β4 with HPV status in CIN.

The onset of proliferative intraepithelial change implies altered tissue kinetics with more rapid escape of neoplastic cells from the basal layers of the tissue as well as loss of

**Figure 5** Immunoperoxidase localisation of the G71 epitope in normal cervix (**a**) and CIN III (**b**). The basal localisation observed in the normal tissue is largely consistent with that observed using other anti-β4 antibodies, although no lateral staining is observed. Note also that the epitope is detected in small blood vessels in the superficial dermis. In CIN III, staining in the basal layer is somewhat weaker, but pericellular reactivity appears in upper cell layers within the lesion with considerable variation in reactivity between individual cells. Magnification: ×495.

differentiated cells from intermediate and superficial layers. This is likely to be accompanied by altered cell polarisation and architecture, intercellular and cell–basement membrane adhesive interactions (Liotta *et al.*, 1991). The presence of β4-containing cells in extrabasal layers of the neoplastic epithelium could imply a more rapid escape from the basal layer of cells bearing the residual hallmarks of the basal cell phenotype, or alternatively it may be that integrin β4 is capable of a function in intercellular organisation other than adhesion to basement membrane. Loss of hemidesmosomes is a prerequisite of cell migration, either in wound healing (Kurpakus, 1991) or tumour invasion (McNutt, 1976). During trophoblast invasion loss of α6β4 occurs from the migrating cell population soon after the loss of adhesion to the villous basement membrane (Aplin, 1993); this contrasts with the persistence of α6β4 in cervical neoplastic cells *in situ* as well as in invasive foci (Carico *et al.*, 1993; Hughes *et al.*, 1994).

The properties of monoclonal antibodies 5B5 and 439-9B show clearly that they recognise the extracellular domain of integrin β4 (Sonnenberg *et al.*, 1991) and immunoprecipitate α6β4 complexes. In addition to the full length polypeptide, these antibodies capture truncated forms of the β4 chain present in both cell lines. The properties of these isoforms are similar to those described previously for C-terminally truncated β4 chains (Falcioni *et al.*, 1988; Kennel *et al.*, 1989; Van Waes *et al.*, 1991; Giancotti *et al.*, 1992). These can also be captured using antibody to α6, confirming their ability to complex stably with a β4 chain. We cannot exclude that some proteolysis of β4 occurred during our experiments since calcium ions were present, and Giancotti *et al.* (1992) have shown that the cytoplasmic domain of the subunit is sensitive to a calcium-dependent protease, calpain. Their evidence suggests that truncated forms of β4 are present along with the full length chain *in vivo*. The immunoprecipitation analysis reported here, in which we have demonstrated that G71 recognises a 200 kDa chain in BeWo cells with precisely the same electrophoretic properties as one recognised by a polyclonal antibody to the C-terminus of human integrin β4, confirms previous evidence (Aplin *et al.*, 1992) that G71 recognises the integrin β4 subunit. In contrast, no subunit was precipitated by G71 from T47D cells in several different experiments. This suggests that different β4 molecular isoforms may be present in different cell types, not all of which are recognised by G71. The G71 epitope is in the extracellular domain of β4 as monitored by light and electron microscopic immunolocalisation in normal amnion epithelial cells (Aplin and Seif, 1985; JD Aplin and DR Garrod, unpublished results). G71 recognises the full length β4 subunit in preference to shorter variants suggesting that its binding site may be near the amino terminus of the molecule and therefore lost during N-terminal proteolytic truncation.

It was therefore of interest to compare the distribution of the G71 and 5B5 epitopes immunohistochemically. Differential expression of β4 isoforms may reflect the heterogeneity of neoplastic cell behaviour and could be relevant to pathophysiology of different phenotypes. The greater heterogeneity of binding of G71 to neoplastic cells suggests that a varying degree of proteolytic or other modification may occur after escape from the basement membrane.

We have shown that α6β4 integrin displays an abnormal distribution with remarkable consistency and in a fashion that reflects the extent and grade of CIN. The appearance of α6β4 in association with neoplastic cells suggests the possibility that it may also occur in detectable quantities in cervical smears. Our data support the view that the analysis of molecular changes occurring in preinvasive conditions offers the hope of improved basic understanding of the disease process as well as the possibility of novel, and perhaps more convenient, approaches to diagnosis.

**Acknowledgements**
We are grateful to Wellbeing and CRC for financial support, and to Dr M Pearson for independent assessment of cervical histology.

### References

ANDERSON GH, BOYES DA, BENEDET JL, LE RICHE JC, MATISIC JP, SUEN KC, WORTH AJ, MILLNER A AND BENNETT OM. (1988). Organisation and results of the cervical cytology screening programme in British Columbia, 1995–1985. *Br. Med. J.*, **296,** 975–978.

ANDREW SM AND JASANI B. (1987). An improved method for the inhibition of endogenous peroxidase non-deleterious to lymphocyte surface markers. Application to immunoperoxidase studies on eosinophil-rich tissue preparations. *Histochem. J.*, **19,** 426–430.

APLIN JD. (1993). Expression of integrin α6β4 by human trophoblast and its loss from extravillous cells. *Placenta*, **14,** 203–215.

APLIN JD AND SEIF MW. (1985). Basally located cell surface component identified using a novel monoclonal antibody technique. *Exp. Cell Res.*, **160,** 550–555.

APLIN JD, SATTAR A AND MOULD AP. (1992). Variant choriocarcinoma (BeWo) cells that differ in adhesion and migration on fibronectin display conserved patterns of integrin expression. *J. Cell Sci.*, **103,** 435–444.

APLIN JD AND CHURCH HJ. (1994). Basement membrane-hemidesmosome interactions. In *Molecular Biology of Desmosomes and Hemidesmosomes*, Garrod DR, Collins J (eds) pp. 87–106. RG Landes: Austin, Texas.

BEHZAD F, JONES CJP, BALL S, ALVARES T AND APLIN JD. (1995). Studies of hemidesmosomes in human amnion: the use of a detergent extraction protocol for compositional and ultrastructural analysis and preparation of a hemidesmosome-enriched fraction from tissue. *Acta Anat.*, **152,** 170–184.

CARICO C, FRENCH D, BUCCI B, FALCIONI R, VECCHIONE A AND MARIANI-CONSTANTINI R. (1993). Integrin β4 expression in the neoplastic progression of cervical epithelium. *Gynecol. Oncol.*, **49,** 61–66.

CARTER WG, KAUP P, GIL SG, GAHR PJ AND WAGNER AE. (1990). Distinct functions for integrins α3β1 in focal adhesions and α6β4/bullous pemphigoid antigen in a new stable anchoring contract of keratinocytes. *J. Cell Biol.*, **111,** 3141–3154.

DE LUCA M, TAMURA RN, KAJIJI S, BONDANZA S, ROSSINO P, CANCEDDA R, MARCHISIO PC AND QUARANTA V. (1990). Polarised integrin mediates human keratinocyte adhesion to basal lamina. *Proc. Natl Acad. Sci. USA*, **87,** 6888–6892.

FALCIONI R, SACCHI A, RESAU J AND KENNEL S. (1988). Monoclonal antibody to human carcinoma-associated protein complex: quantitation in normal and tumor tissue. *Cancer Res.*, **48,** 816–821.

FRIXEN UH, BEHRENS J, SACHS M, EBERLE G, VOSS B, WARDA A, LOCHNER D AND BIRCHMEIER W. (1991). E-cadherin-mediated cell–cell adhesion prevents invasiveness of human carcinoma cells. *J. Cell Biol.*, **113,** 173–185.

GIANCOTTI FG AND RUOSLAHTI E. (1990). Elevated levels of the α5β1 fibronectin receptor suppress the transformed phenotype of chinese hamster ovary cells. *Cell*, **60,** 849–859.

GIANCOTTI FG, STEPP MA, SUZUKI S, ENGVALL E AND RUOSLAHTI E. (1992). Proteolytic processing of endogenous and recombinant β4 integrin subunit. *J. Cell Biol.*, **118,** 951–959.

HODIVALA KJ, PEI XF, LIU QY, JONES PH, RYTINA ER, GILBERT C, SINGER A AND WATT FM. (1994). Integrin expression and function in HPV 16-immortalised human keratinocytes in the presence or absence of v-Ha-ras. Comparison with cervical intraepithelial neoplasia. *Oncogene*, **9,** 943–948.

HOGERVORST F, KUIKMAN I, VON DEM BORNE, AEG AND SONNENBERG A. (1990). Cloning and sequence analysis of β4 cDNA: an integrin subunit that contains a unique 118 kDa cytoplasmic domain. *EMBO J.*, **9,** 765–770.

HUGHES DE, REBELLO G AND AL-NAFUSSI A. (1994). Integrin expression in squamous neoplasia of the cervix. *J. Pathol.*, **173,** 97–104.

HYNES RO. (1992). Integrins: versatility, modulation and signalling in cell adhesion. *Cell*, **69,** 11–25.

KENNEL SJ, FOOTE LJ, FALCIONI R, SONNENBERG A, STRINGER CD, CROUSE C AND HEMLER ME. (1989). Analysis of the tumor-associated antigen TSP-180: identity with α6β4 in the integrin superfamily. *J. Biol. Chem.*, **264,** 15515–15521.

KURPAKUS MA, QUARANTA V AND JONES JCR. (1991). Surface relocation of α6β4 integrins and assembly of hemidesmosomes in an *in vitro* model of wound healing. *J. Cell Biol.*, **115,** 1737–1750.

LEE EC, LOTZ MM, STEELE GD AND MERCURIO AM. (1992). The integrin α6β4 is a laminin receptor. *J. Cell Biol.*, **117,** 671–678.

LIOTTA LA, STEEG PS AND STETLER-STEVENSON WG. (1991). Cancer metastasis and angiogenesis: an imbalance of positive and negative regulation. *Cell*, **64,** 327–336.

LOTZ MM, KOZELIUS CA. AND MERCURIO AM. (1990). Human colon carcinoma cells use multiple receptors to adhere to laminin: involvement of α6β4 and α2β1 integrins. *Cell Regul.*, **1**, 249-257.

MCNUTT NS. (1976). Ultrastructural comparison of the interface between epithelium and stroma in basal cell carcinoma and control human skin. *Lab. Invest.*, **35,** 132–142.

NIESSEN CM, HOGERVORST F, JASPARS LH, DE MELKER AA, DELWEL GO, HULSMAN EHM, KUIKMAN I AND SONNENBERG A. (1994). The α6β4 integrin is a receptor for both laminin and kalinin. *Exp. Cell Res.*, **211,** 360–367.

PHILLIPS RJ, APLIN JD AND LAKE BD. (1994). Antigenic expression of integrin α6β4 in junctional epidermolysis bullosa. *Histopathology*, **24,** 571–576.

PLANTEFABER LC AND HYNES RO. (1989). Changes in integrin receptors on oncogenically transformed cells. *Cell*, **56,** 281–290.

POTTS AJ, CROALL DE AND HEMLER ME. (1994). Proteolytic cleavage of the integrin β4 subunit. *Exp. Cell Res.*, **212,** 2–9.

RISINGER JI, BERCHUCK A, KOHLER MF AND BOYD J. (1994). Mutations of the E-cadherin gene in human gynecologic cancers. *Nature Genet.*, **7,** 98–102.

SONNENBERG A AND LINDERS CJT. (1990). The α6β1 (VLA-6) and α6β4 protein complexes: tissue distribution and biochemical properties. *J. Cell Sci.*, **96,** 207–217.

SONNENBERG A, CALAFAT J, JANSSEN H, DAAMS H, VAN DER RAAIJ-HEMLER LMH, FALCIONI R, KENNEL SJ, APLIN JD, BAKER J, LOIZIDOU M AND GARROD D. (1991). Integrin α6β4 complex is located in hemidesmosomes suggesting a major role in epidermal cell–basement membrane adhesion. *J. Cell Biol.*, **113,** 907–917.

STEPP MA, SPURR-MICHAUD S, TISDALE A AND GIPSON IK. (1990). α6β4 integrin heterodimer is a component of hemidesmosomes. *Proc. Natl Acad. Sci. USA*, **87,** 8970–8974.

SUZUKI S AND NAITOH Y. (1990). Amino acid sequence of a novel integrin β4 subunit and primary expression of the mRNA in epithelial cells. *EMBO J.*, **9,** 757–763.

SYMINGTON B. (1990). Fibronectin receptor overexpression and loss of transformed phenotype in a stable variant of the K562 cell line. *Cell Regul.*, **1,** 637–648.

TAMURA RN, ROZZO C, STARR L, CHAMBERSM J, REICHARDT LF, COOPER HM AND QUARANTA V. (1990). Epithelial integrin α6β4: Complete primary structure of α6 and variant forms of β4. *J. Cell Biol.* **111,** 1593–1604.

TIDMAN MJ, APLIN JD AND MACDONALD DM. (1990). Abnormal expression of the G71 antigen at the epidermal basement membrane in basal cell carcinoma. *Cancer*, **65,** 1955–1959.

VAN WAES C KOZARSKY KF, WARREN AB, KIDD L, PAUGH D, LIEBERT M AND CAREY TF. (1991). The A9 antigen associated with aggressive human squamous carcinoma is structurally and functionally similar to the newly defined integrin α6β4. *Cancer Res.*, **51,** 2395–2402.

VIDAL F, ABERDAM D, MIQUEL C, CHRISTIANO AM, PULKKINEN L, UITTO J, ORTONNE JP AND MENEGUZZI G. (1995). Integrin β4 mutations associated with junctional epidermolysis bullosa with pyloric atresia. *Nature Genet.*, **10,** 229–234.